# False-Positive Detection of Haemophilus influenzae by a Multiplex PCR Panel in a Neonate With Late-Onset Group B Streptococcal Bacteremia

**DOI:** 10.7759/cureus.86997

**Published:** 2025-06-29

**Authors:** Koji Yokoyama, Ayako Okamoto, Misako Ohkusu, Naruhiko Ishiwada

**Affiliations:** 1 Department of Pediatrics, Japanese Red Cross Wakayama Medical Center, Wakayama, JPN; 2 Department of Infectious Diseases, Medical Mycology Research Center, Chiba University, Chiba, JPN

**Keywords:** false-positive reactions, filmarray me panel, group b streptococcus (gbs), haemophilus influenzae serotype b, multiplex polymerase chain reaction

## Abstract

Here, we report the case of a term neonate with late-onset Group B Streptococcal (GBS) bacteremia in whom the FilmArray® Meningitis/Encephalitis (ME) panel (BioFire Diagnostics, Salt Lake City, UT, US) falsely detected *Haemophilus influenzae *in the cerebrospinal fluid (CSF). Although the CSF showed mild pleocytosis and elevated protein, there were no clinical signs of bacterial meningitis. Confirmatory testing, including multiplex PCR targeting *H. influenzae* (siaT), GBS (cfb), and 16S rRNA, as well as CSF culture, yielded negative results. Repeat FilmArray testing under controlled conditions was also negative. A manufacturer's investigation revealed borderline amplification of only one of the *H. influenzae* targets, which was interpreted algorithmically as positive. This discordance highlights a key limitation of multiplex PCR panels: the lack of transparency in signal interpretation and threshold settings. The patient demonstrated no immunodeficiency and remained clinically stable during a 10-month follow-up. This case underscores the importance of confirmatory testing when panel results contradict the clinical picture and advocates for improved algorithmic clarity and inter-laboratory validation. Increased awareness of such diagnostic pitfalls is essential, particularly in neonatal settings where unnecessary antimicrobial interventions may pose additional risks.

## Introduction

The FilmArray® Meningitis/Encephalitis (ME) panel (BioFire Diagnostics, Salt Lake City, UT, US) enables rapid multiplex PCR-based detection of 14 pathogens directly from the cerebrospinal fluid (CSF) and is widely used for diagnosing meningitis and encephalitis. Its analytical performance, including turnaround time and target coverage, has been evaluated in multicenter validation studies [1]. Furthermore, a systematic review and meta-analysis confirmed that the assay has high pooled sensitivity and specificity across diverse clinical settings [2]. Multiplex PCR simultaneously detects multiple pathogens in a single reaction, enabling rapid diagnosis; however, false positives can lead to unnecessary treatments, which may pose particular risks in neonates. Despite these strengths, false-positive results, particularly for *Haemophilus influenzae*, remain a critical concern. Here, we present a neonatal case with a false-positive *H. influenzae* result and discuss the clinical and laboratory implications.

## Case presentation

A male term neonate, born at 39 weeks 2 days via vaginal delivery, received ampicillin at the onset of labor due to maternal carriage of Group B Streptococcus (GBS). At 21 days of life, the patient developed fever and lethargy, prompting evaluation for late-onset sepsis. He was an only child with no recent exposure to other young children. A blood culture was positive for GBS. Although maternal colonization was identified at 36 weeks of gestation, a throat swab obtained from the neonate at birth was negative. Initial CSF analysis revealed mildly elevated cell counts (total 103/μL; 53 mononuclear, 50 polymorphonuclear), likely due to traumatic lumbar puncture, with visible blood contamination. CSF protein was elevated (142 mg/dL), while glucose was within the normal range (51 mg/dL; concurrent blood glucose 90 mg/dL). These findings are inconsistent with typical bacterial meningitis; however, the FilmArray ME panel returned a positive result for *H. influenzae*. Because of the lack of definitive signs of meningitis and discordance with the panel result, the CSF specimen was reanalyzed at an independent facility. Multiplex PCR targeting *H. influenzae* (siaT), GBS (cfb), and 16S rRNA failed to detect bacterial DNA, and the CSF culture was negative. The same CSF specimen was retested with the FilmArray ME panel under controlled conditions at an independent facility. To minimize contamination risk, the sample was handled in a class II biosafety cabinet using sterile, single-use consumables, and all procedures were performed by trained personnel following the manufacturer’s protocols. The timeline of the clinical events and diagnostics is presented in Table 1.

**Table 1:** Clinical and diagnostic timeline of the patient Chronological summary of symptoms, lumbar puncture, FilmArray ME panel testing, confirmatory PCR and culture, and treatments. Given the clinical context and discordant test results, therapeutic decisions were based primarily on blood culture results rather than initial CSF findings. ABPC: ampicillin, CTX: cefotaxime.

Day of life	Clinical event	Diagnostic or therapeutic action
0	Term birth	GBS-positive mother, intrapartum antibiotics administered
19	Fever, poor feeding	Hospital admission, empirical ABPC started
20	Suspected severe infection	Initial FilmArray ME panel detected GBS (blood) and *H. influenzae* (CSF). Combination therapy with ABPC and CTX continued
22-28	Clinical improvement	Monotherapy with ABPC continued
29	Discharge	Stable, afebrile, feeding well

**Figure 1:**
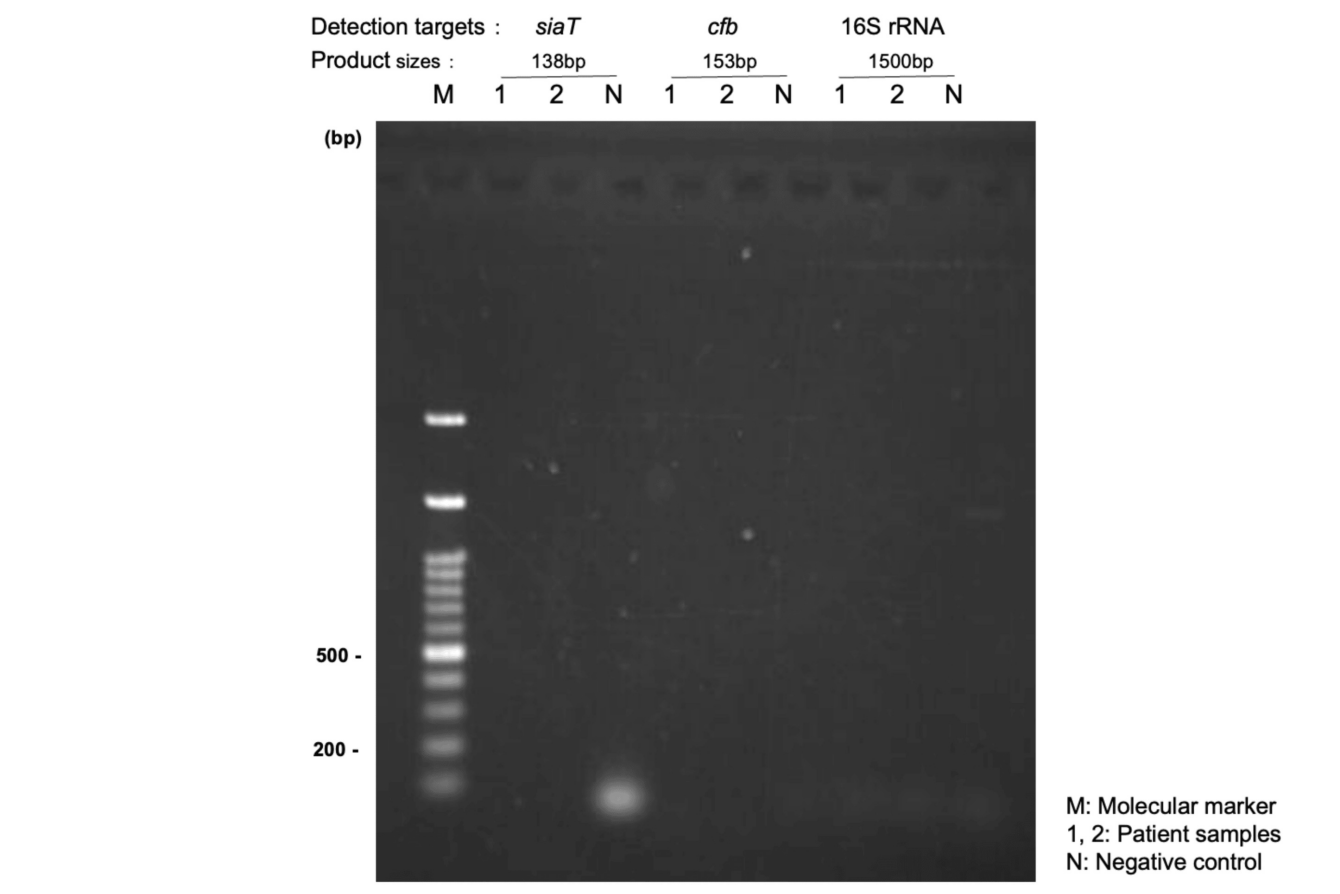
Gel electrophoresis results for the PCR targeting specific bacterial genes DNA extracted from the cerebrospinal fluid (CSF) was amplified with primers specific for *Haemophilus influenzae* (siaT), Group B Streptococcus (GBS, cfb), and universal 16S rRNA. Expected product sizes: siaT, 138 bp; cfb, 153 bp; 16S rRNA, ~1.5 kbp. No amplification was observed. Lane M, marker; Lane 1, neat (undiluted) CSF; Lane 2, 1:2 diluted CSF; Lane N, negative control.

No amplification of any targets was observed on gel electrophoresis (Figure 1).

The manufacturer concluded that the initial detection of *H. influenzae* likely resulted from low-level environmental contamination during sample handling. Immunological evaluations, including immunoglobulin and complement levels, were within normal limits. The patient had no signs of immunodeficiency during the 10 months of follow-up.

The parents of the patient provided informed consent for the publication of this case, and the study was approved by the Institutional Review Board of the Japanese Red Cross Wakayama Medical Center (Approval No. 1481).

## Discussion

We describe a false-positive *H. influenzae* result on the FilmArray ME panel without supporting clinical or laboratory evidence. A summary of the CSF test results is provided in Table 2.

**Table 2:** Summary of CSF diagnostic test results Results of the initial FilmArray ME panel (positive for *Haemophilus influenzae*), confirmatory polymerase chain reaction (PCR) assays targeting *H. influenzae* (siaT), Group B Streptococcus (GBS, cfb), and universal 16S rRNA, and independent cerebrospinal fluid (CSF) cultures. GBS: Group B Streptococcus, CSF: cerebrospinal fluid, PCR: polymerase chain reaction.

Test method	Sample	Target organism(s)	Result	Notes
FilmArray ME panel	CSF	H. influenzae	Positive	Only Hinf1 target showed delayed amplification; Hinf2 negative
CSF culture (initial)	CSF	–	Negative	Sample contaminated with blood
PCR (Chiba Univ, manual)	CSF	siaT, cfb, 16S rRNA	Negative	All targets negative; confirmatory testing performed

Although the overall false-positive rate for *H. influenzae* on this panel is <0.1% [3], a previous study reported that 77.8% (14/18) of *H. influenzae*-positive cases were not confirmed by qPCR or culture [4]. Other retrospective studies also question the alignment of FilmArray results with discharge diagnoses in low-prevalence settings [5]. Environmental contamination during specimen handling may contribute to false-positive results in highly sensitive assays [6,7]. Despite standard decontamination protocols, such occurrences persist in clinical practice, particularly when handling highly sensitive molecular assays in open systems. Current diagnostic platforms often lack integrated contamination safeguards and transparent signal interpretation tools, which may contribute to rare but consequential false-positive results. In this case, the manufacturer found borderline amplification of only one target (Hinf1), while the confirmatory target (Hinf2) was negative. Nonetheless, the system algorithm reported a positive result, consistent with previously noted limitations in qPCR transparency and cycle threshold (Ct) data interpretation [8,9]. Immunodeficiency was unlikely given the patient's normal immunologic profile and uneventful course. Clinicians should remain cautious when test results conflict with the clinical picture, particularly in neonates for whom overtreatment poses additional risks [10]. Future diagnostic platforms should offer sealed cartridges, automated preprocessing, and visible assay-level confidence metrics to reduce unnecessary interventions [11,12]. Emerging methods, including metagenomic sequencing and microbial cell-free DNA testing, may complement standard diagnostics when dealing with complex central nervous system infections [13]. This case underscores the importance of inter-laboratory validation and careful interpretation when laboratory findings are inconsistent with clinical presentation. Given the clinical implications of false-positive syndromic panel results, systematic reporting and data sharing of such cases across institutions may help refine diagnostic criteria and guide future algorithm development.

## Conclusions

We report a case in which the FilmArray ME panel falsely detected *H. influenzae* in the CSF of a neonate with confirmed GBS bacteremia. The false-positive result was overturned by independent molecular and culture-based testing. This case highlights the importance of confirmatory testing and critical clinical judgment when interpreting syndromic panel results, particularly in neonates for whom overdiagnosis may lead to unnecessary interventions.
